# Assessment of the Quality of Reporting in Abstracts of Randomized Controlled Trials Published in Five Leading Chinese Medical Journals

**DOI:** 10.1371/journal.pone.0011926

**Published:** 2010-08-02

**Authors:** Yaolong Chen, Jing Li, Changlin Ai, Yurong Duan, Ling Wang, Mingming Zhang, Sally Hopewell

**Affiliations:** 1 Chinese Cochrane Centre, West China Hospital, Sichuan University, Chengdu, Sichuan, China; 2 Evidence-Based Medicine Center, School of Basic Medical Sciences, Lanzhou University, Lanzhou, Gansu, China; 3 UK Cochrane Centre, Oxford, United Kingdom; Pennington Biomedical Research Center, United States of America

## Abstract

**Background:**

Clear, transparent and sufficiently detailed abstracts of randomized trials (RCTs), published in journal articles are important because readers will often base their initial assessment of a trial on such information. However, little is known about the quality of reporting in abstracts of RCTs published in medical journals in China.

**Methods:**

We identified RCTs abstracts from 5 five leading Chinese medical journals published between 1998 and 2007 and indexed in MEDLINE. We assessed the quality of reporting of these abstracts based on the Consolidated Standards of Reporting Trials (CONSORT) abstract checklist. We also sought to identify whether any differences exist in reporting between the Chinese and English language version of the same abstract.

**Results:**

We identified 332 RCT abstracts eligible for examination. Overall, the abstracts we examined reported 0–8 items as designated in the CONSORT checklist. On average, three items were reported per abstract. Details of the interventions (288/332; 87%), the number of participants randomized (216/332; 65%) and study objectives (109/332; 33%) were the top three items reported. Only two RCT abstracts reported details of trial registration, no abstracts reported the method of allocation concealment and only one mentioned specifically who was blinded. In terms of the proportion of RCT abstracts fulfilling a criterion, the absolute difference (percentage points) between the Chinese and English abstracts was 10% (ranging from 0 to 25%) on average, per item.

**Conclusions:**

The quality of reporting in abstracts of RCTs published in Chinese medical journals needs to be improved. We hope that the introduction and endorsement of the CONSORT for Abstracts guidelines by journals reporting RCTs will lead to improvements in the quality of reporting.

## Introduction

There are more than 1200 biomedical journals in China [1], which publish thousands of randomized controlled trials (RCTs) each year. Clear, transparent, and sufficiently detailed abstracts of RCTs are more important particularly in China and other developing countries where researchers and health professionals often use an abstract to decide whether to seek more information about a trial; or may have access to the abstracts only [2]. Previous studies have attempted to assess the quality of reporting in abstracts of clinical studies [3] or to assess the quality of reporting in abstracts of RCTs published in English language journals [4]. The aim of our study is to assess the quality of reporting in abstracts of RCTs published in five leading Chinese medical journals using the recent CONSORT (Consolidated Standards of Reporting Trials) checklist for reporting abstracts of RCTs.

## Materials and Methods

### 1. Selection of journals and sample of abstracts of RCTs

We selected five leading Chinese medical journals indexed in MEDLINE from 1998 to 2007 with top ranking impact factor (IF) in each field based on the “2007 data for Sci-tech Journal Citation Reports of China”(Table 1) and with both English and Chinese abstracts: the National Medical Journal of China, Chinese Journal of Internal Medicine, Chinese Journal of Obstetrics and Gynecology, Chinese Journal of Pediatrics, and the Chinese Journal of Surgery.

**Table 1:** Number of abstracts identified and included from five Chinese medical journals.

Name of journal	Impact factor	Total citations	Number of RCTs	Percent (%)
Chinese Journal of Pediatrics	1.347	2909	33	9.9%
Chinese Journal of Obstetrics and Gynecology	1.121	2719	50	15.1%
National Medical Journal of China	1.091	3792	121	36.5%
Chinese Journal of Internal Medicine	0.903	2409	68	20.5%
Chinese Journal of Surgery	0.963	3222	60	18%
Total			332	100%

Data of impact factor and total citations are from the China Journal Citation Reports (CJCR) in 2007.

The CBM (Chinese Biomedical Database) disk database was searched using an extended version of the Cochrane Highly Sensitive Search Strategy [5] (Table 2) to identify RCTs (searched April, 2008) for Chinese abstracts in each of the five leading journals. Based on the citation of each RCT, we hand searched these five printed journals for the English abstracts. We included all publications reporting RCTs,which was defined as a trial where the allocation of participants to interventions was described by the words random, randomly, randomized, or randomization in all disease areas and all types of interventions, dealing with patients or volunteers.

**Table 2:** Search strategy to identify RCTs in five leading Chinese medical journals.

1 random$
2 randomized controlled trial/
3 randomized controlled trial$.pt.
4 double blind
5 double blind method/
6 single blind
7 single blind method/
8 triple blind
9 blind$
10 or/1–9
11 10/limit:animal
12 10 not 11)

### 2. Evaluation Method

We assessed the quality of reporting in abstracts of RCTs based on the CONSORT for Abstracts checklist [6]. Refined items from the CONSORT for Abstracts are presented in the Table 3. We assessed each item as “reported” or “not reported” according to whether the author reported all the contents listed in the refined items or not. We checked for any differences in reporting between the Chinese and English language version of the same abstract.

**Table 3:** Adapted CONSORT for Abstracts checklist items reported in the 332 journal abstracts of RCTs.

Items	Description	Refined items	No. reported Chinese abstracts (n = 332) (%)	No. reported English abstracts (n = 332) (%)
Title	Identification of the study as randomized		34 (10%)	47 (14%)
Authors*	Contact details for the corresponding author		-	-
Trial design	Description of the trial design		19 (6%)	10 (3%)
Methods				
Participants	Eligibility criteria for participants and the settings where the data were collected		9 (3%)	7 (2%)
		Eligibility criteria for participants	148(45%)	120 (36%)
		Eligibility criteria for settings	15(5%)	13(4%)
Interventions	Interventions intended for each group		288 (87%)	310 (93%)
Objective	Specific objective or hypothesis		109 (33%)	103 (31%)
Outcome	Clearly defined primary outcome for this report	0	0	5 (2%)
Randomization	How participants were allocated to interventions		0	0
		Description of the method for assigning participants	10(3%)	1 (0%)
		Description of allocation concealment	0	0
Blinding (masking)	Whether or not participants, care givers, and those assessing the outcomes were blinded to group assignment		0	0
		Description of whether or not using blinding	49 (15%)	48 (14.%)
		Description of who were blinded	0	1 (0%)
Results				
Numbers randomized	Number of participants randomized to each group		216 (65%)	214 (65%)
Recruitment*	Trial status	-	-	-
Numbers analyzed	Number of participants analyzed in each group		50 (15%)	42 (13%)
Outcome	For the primary outcome, a result for each group and the estimated effect size and its precision		0	0
		Result for each group	135 (41%)	159(48%)
		Estimated effect size of a result for each group	9 (3%)	4 (1%)
		Precision of the estimate	6 (2%)	6 (2%)
Harms	Important adverse events or side effects		30 (9%)	33 (10%)
Conclusions	General interpretation of the results		22 (7%)	15 (5%)
Trial registration	Registration number and name of trial register		2 (1%)	2 (1%)
Funding	Source of funding		86 (26%)	86 (26%)

*Authors and Recruitment are most specific to conference abstracts, and were not included.

We determined the number and proportion (%) of RCT abstracts that reported each of the CONSORT for Abstracts checklist items. Four assessors (YL Chen, CL Ai, L Wang and YR Duan) independently rated each RCT abstract and inter-rater agreement for each checklist item was determined using the Kappa statistic. Overall, this produced good agreement between the reviewers (0.7). Discrepancies were resolved by consensus and or discussed. Data analysis was carried out using Excel 2003, Epidata 3.02 and Stata version 9.0.

## Results

### 1. Quality of reporting in Chinese abstracts

**Figure 1:**
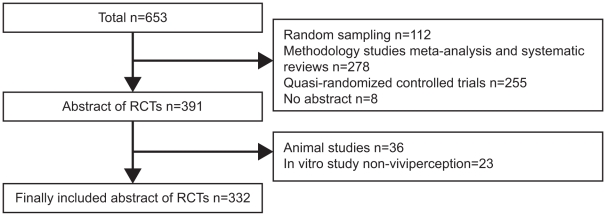
Identification of RCT abstracts from the Chinese Biomedical Database.

We identified 692 abstracts of RCTs during our initial search and subsequently determined that 332 pairs were eligible for analysis based on our criteria of findings being reported both Chinese and English (Figure 1). We have presented the results of our findings in Table 3. Overall only 10% (34/332) of the abstracts could be identified as an RCT based on the title. No abstracts mentioned the method of allocation concealment and, while 49 (15%) RCT abstracts reported on blinding, only one indentified who was blinded. Although 65 (20%) of the abstracts mentioned adverse events or side effects, less than 10% reported specific symptoms (n = 20) or rates (n = 26). Lastly, 246 (75%) abstracts did not report their sources of funding.

### 2. Comparison the quality of reporting between the Chinese with English abstracts

The results of the English language version of the same RCT abstracts are also presented in Table 3. In terms of the proportion of RCT abstracts fulfilling a criterion, the absolute difference (percentage points) between the Chinese and English abstracts was 10% (ranging from 0 to 25%) on average, per item. Some items such as details of the primary outcome, were only reported in the English version of the abstract. Some items were complementary between the Chinese and English abstracts, for example, some Chinese abstracts only reported the “numbers randomized” while their corresponding English abstracts only reported the “numbers analyzed”.

## Discussion

### 1. General quality of reporting in abstracts and its possible reasons

Our study indicates that the quality of reporting in abstracts of RCTs published in these five leading Chinese medical journals is insufficient. Overall, the average number of reported items in each RCT abstract, based on the CONSORT for Abstracts checklist, was three. None of the abstracts adequately reported the key features of the trial design, including the method of generating the allocation sequence, concealment of the allocation, or the estimated effect size and its precision for the primary outcome. Less than half of the RCT abstracts reported the study as randomized in the title, the number of participants analysed, details of harms, trial registration and source of funding.

Berwanger and colleagues [7] evaluated the quality of reporting in 227 abstracts describing RCTs published in the New England Journal of Medicine, JAMA, the BMJ and the Lancet in 2006. In their paper, the authors identified that details regarding methodological quality were also poorly reported as only one RCT abstract described the method of allocation concealment; 21 (9%) clearly specified who was blinded; 51 (23%) described intention-to-treat analysis; and 32 (14%) outlined losses to follow-up.

In the mid-1990s, Anrong [8] and Bili [9] reported that the quality of abstracts in Chinese medical journals was poor. They suggested that poor abstract reporting may indicate suboptimal study design, inadequate reporting, or both. Previous work has shown that abstracts frequently under report key features of study design [10]–[11] and omit important results. It is suggested that the poor quality of reporting of Chinese abstracts may also be an indication of poor reporting in full-text RCTs in China [12]–[18]. When an item isn't reported in the full publication, it is likely to also be missing from the abstract. A lack of detailed requirement for abstracts in a journal's ‘Instruction for Authors’ is also a potential problem. Although most of the journals included in our study have adopted structured abstracts, the structured format alone is not sufficient to guide authors regarding their content. Thus, some of the most salient information might be missed if no detailed instructions for each heading are given. Finally, a lack of sufficient knowledge of research methodology might also attribute to the poor quality of reporting.

Abstracts should contain the most important information and accurate study results relevant to clinical practice. While we did find that most abstracts reported the purported “study benefits,” we also observed that the primary outcome, the estimated effect size, and the precision and harms of the study were rarely reported. Cumulatively, this lack of information hampers readers in making clinical decisions [19]–[20] and presents clinicians many difficulties when trying to evaluate the benefits and harms of any intervention. Unfortunately, only two RCT abstracts reported details of trial registration.

While, not included in our study, the Chinese Journal of Evidence-Based Medicine recently introduced the CONSORT for Abstracts guidelines into its ‘Instructions for Authors’ [21]. The application of the CONSORT Statement has demonstrated benefits in improving the quality of reporting in abstracts [22]–[24], and we hope that it will do the same with the introduction of CONSORT for Abstracts. We very much hope that the five journals included in our study will also consider including such guideline to their ‘Instructions for Authors’.

### 2. Limitations

This study has several limitations, firstly, we did not take a random sample of all RCT abstracts published in Chinese medical journals and therefore our findings may not reflect all abstracts of RCTs published in China or in other countries. Secondly, we did not compare abstracts with their corresponding full articles, However, previous studies have shown that the quality of reporting of the full-text is also poor in the same medical journals [12]–[18]. We further wish to point out that the WHO International Clinical Trial Register Platform only approved The Chinese Clinical Trial Register (ChiCTR) in 2007. Thus, it may well be that many researchers do not know the importance of prospective trial registration [25]. While not included in our current study, the Chinese Journal of Evidence-Based Medicine recently introduced the CONSORT for Abstracts guidelines into its ‘Instructions for Authors’ [21]. This adoption has improved the quality of reporting in other journals [22]–[24] and it is our sincere hope that the five journals included in our study, as well as other Chinese journals, adopt the CONSORT statement for Abstracts into their ‘Instructions for Authors.’ We believe that this adoption will markedly improve both the interpretation and practice of medicine in China in the future.

### Conclusions

In summary, the quality of reporting in abstracts of RCTs published in these five leading Chinese medical journals requires substantial improvement to meet the recommendations set out in CONSORT for Abstracts guidelines. With the publication of CONSORT for Abstracts checklist, we suggest that Chinese medical journals should adopt these recommendations and do more to ensure that authors apply to meet internationally agreed standards, thereby allowing the conduct of their studies to be monitored and improved.
